# Hepatocyte Aggregate Formation on Chitin-Based Anisotropic Microstructures of Butterfly Wings

**DOI:** 10.3390/biomimetics3010002

**Published:** 2018-01-18

**Authors:** Abdelrahman Elbaz, Bingbing Gao, Zhenzhu He, Zhongze Gu

**Affiliations:** 1 State Key Laboratory of Bioelectronics, School of Biological Science and Medical Engineering, Southeast University, Nanjing 210096, China; chem.egy@gmail.com (A.E.); 230139435@seu.edu.cn (B.G.); 230169448@seu.edu.cn (Z.H.); 2 National Demonstration Center for Experimental Biomedical Engineering Education, Southeast University, Nanjing 210096, China; 3 Laboratory of Environment and Biosafety, Research Institute of Southeast University in Suzhou, Suzhou 215123, China

**Keywords:** organotypic culture models, cell aggregate, HepG2, 3D scaffolds, anisotropic microstructure

## Abstract

Scaffold nanotopography plays the most significant role in the mimicry of the in vivo microenvironment of the hepatocytes. Several attempts have been made to develop methods and substrates suited to growing hepatocytes into aggregates. Functional biomaterials, particularly biodegradable polymers, have been used in several studies aimed to develop improved scaffolds with ordered geometry and nanofibrous architecture for tissue engineering. However, there are still some limitation in their fabrication: it is not cost-efficient, is time-consuming, and exhibits some technological complications. The synthetic scaffolds are usually non-biodegradable and can be non-biocompatible compared to the naturally derived biomaterials. Here, we utilized a simple, cost-effective, and green method with two-step chemical treatment to get more selected hydrophilic butterfly wings from *Morpho menelaus*, *Papilio ulysses telegonus*, and *Ornithoptera croesus lydius* as a chitin-based natural scaffolds to growing hepatocyte aggregates. We established a three-dimensional (3D) in vitro model for culture of HepG2 cells and aggregate formation that maintained the hepatocytes function on these natural anisotropic microstructures. Cells cultured on these substrates show higher viability than those cultured on a two-dimensional (2D) culture plate. Methylthiazolyldiphenyl-tetrazolium bromide (MTT) assay results revealed excellent viability of HepG2 cells on *P. u. telegonus* wings (fibrous area). The results also demonstrated appropriate cell activity, cell retention, and stable and functional expression in terms of albumin secretion and urea synthesis activity compared to the 2D monolayer culture of hepatocytes on the culture dish surface. With a slightly different degree, the other substrates also shown similar results. We anticipate that these natural anisotropic, biodegradable, and biocompatible substrates can maintain long-term hepatic culture as an in vitro 3D model for potential therapeutic applications and regenerative tissue applications. The model presented here provides a feasible alternative to the synthetic scaffolds and is expected to be more reliable for 3D organotypic liver culture models based on such scaffolds.

## 1. Introduction

The three-dimensional order (3D) microenvironment complexity contributes to regulate tumor growth in vivo [1,2,3,4]. In the liver tissue, hepatocytes are shaped in a single cord-like aggregate and connected by adjacent sinusoids [5,6,7,8]. Studies have been shown that hepatocytes cultured as spheroids have the ability to maintain long-term hepatic function and promote cell–cell communication and gap junction [9,10]. Researchers have made several attempts to develop methods and substrates suited to growing hepatocytes into aggregates in order to mimic the in vivo liver microenvironment. However, most tumorigenic cell culture studies involve cells cultured on rigid and wholly flat surfaces [11,12,13,14,15,16]. Although the use of cell culture plates and wells is efficient, convenient, and lends itself to high-throughput processing, cell morphology and function can vary greatly based on the used extracellular matrix, which can potentially skew the results of growth, proliferation, differentiation, and chemo-/radiotherapy studies [17,18,19,20]. Moreover, as tumor formation and growth stems from specific gene expression profiles, a cancer cell line that may have undergone multiple passages may produce biased results. It would be more accurate to mimic cancer cell regulation and biological pathways in a 3D setting [21,22,23,24]. Additionally, hepatocytes cultured on two-dimensional (2D) monolayer models also have some limitations as shown in a drug screening study where cultured hepatocytes resulted in a decrease of liver-specific functionality and gene expression, and viability [25,26,27].

In recent years, rapid prototyping computer-controlled methods have been useful for the manufacturing of scaffolds with ordered geometry [25,26,27,28,29,30,31]. Depending on the production process, scaffolds with different design can be obtained with random or tailored pore distribution. Cell culture scaffolds can be made of synthetic materials (e.g., polylactide), or composed of natural matrix components (e.g., Matrigel^TM^, laminin, or collagen) [32,33,34,35,36]. Electrospun fibers have been extensively studied as a component of cell culture scaffolding; a fibrous extracellular matrix (ECM) with microscale interconnected pores is essential to supply nutrients and transport oxygen for cell growth [37,38,39,40]. It has been also reported that self-assembled peptide nanofibers as scaffolds show an enhancement of the hepatic function and promotion of a malignant phenotype in HepG2 cells [41]. However, synthetic scaffolds are usually non-biodegradable and can be non-biocompatible, and it may be difficult, costly, and time-consuming to cast animal-derived biomaterials into the desired scaffold structure [42,43]. It has been reported, that chitin-based composite biomaterials have a biomimetic potential as biocompatible materials in an environmentally sustainable fashion [44].

Here, we report the use of a natural material with distinct nanotopography (i.e., the butterfly wings of *Morpho menelaus*, *Papilio ulysses telegonus*, and *Ornithoptera croesus lydius*), as scaffolds inherited from their natural 3D microstructure, for growing hepatocyte aggregate. A simple, cost-effective, and green method with two-step chemical treatment was utilized to obtain more hydrophilic chitin-based natural scaffolds from butterfly wings. These substrates were previously developed by us and revealed a high degree of alignment of NIH-3T3 fibroblasts along the direction of the ridges. These natural substrates show an important role in the final organotypic model and formation of hepatocyte aggregates, and in maintaining hepatocytes functions as well [45]. We established an in vitro 3D model for hepatocyte growth and aggregate formation maintaining the function of the hepatocytes based on the natural anisotropic microstructures derived from butterfly wings. Methylthiazolyldiphenyl-tetrazolium bromide (MTT) assay results demonstrated the appropriate viability of HepG2 cells on these butterfly wings. HepG2 aggregates on *P. u. telegonus* wings (fibrous area) revealed excellent cell activity, cell retention, and stable and functional expression in terms of albumin secretion and urea synthesis activity compared to hepatocytes cultured on a 2D monolayer model. Other substrates also showed similar results with slightly different degrees. We anticipate that these natural, biodegradable, and biocompatible substrates are suitable to maintain a 3D hepatic microenvironment for potential applications in tissue regeneration and therapeutics. The model presented here provides a feasible alternative to the synthetic scaffolds and is expected to be more reliable for 3D organotypic liver culture models based on 2D scaffolds.

## 2. Materials and Methods

### 2.1. Materials

*M. menelaus*, *P. u. telegonus*, and *O. c. lydius* butterflies were purchased from Dieyu Company (Shanghai, China). Sodium hydroxide (NaOH) and hydrochloric acid (HCl) were purchased from Aladdin Reagent (Shanghai, China). Albumin secretion was assessed by enzyme-linked immunosorbent assay (ELISA) using a commercially available rat albumin quantitation kit (Nanjing Jiancheng Bioengineering Institute, Nanjing, China), while urea levels were assayed with commercially available related kits (Nanjing Jiancheng Bioengineering Institute). All reagents and solvents were analytical grade and were used without further purification. Double-distilled water was used for all experiments [46].

### 2.2. Apparatus 

To hydrophilize the butterfly wings, we used a plasma cleaner (DT-01; SZ-Omega Ltd., Suzhou, China). A field emission scanning electron microscope (JSM-6360LV, JEOL USA Inc., Peabody, MA, USA) equipped with a JED2300 energy-dispersive X-ray spectroscopy (EDS) system (Zeiss Ultra Plus, Zeiss, Jena, Germany) and a scanning electron microscope (S-3000N, Hitachi Ltd., Tokyo, Japan) were used to observe the wing microstructure and the morphology of the cells cultured on the wings. A Quant microplate spectrophotometer (Elx-800, BioTek Instrument Inc., Winooski, VT, USA) was used to measure the absorbance.

### 2.3. Methods

#### 2.3.1. Treatment of Butterfly Wings

Naturally, the butterfly wings are strongly hydrophobic on both the dorsal and ventral sides in order to avoid the influences from rain or other natural disasters. Thus, these wings could not be immersed in any aqueous solutions, precluding any experiments on these wings. In addition, beside chitin, these wings also are constituted by proteins, some pigments and other inorganic salts adopted from the environment that should be removed to obtain a more biocompatible compound for cell culture. Briefly, the scale lumen was pumped to a vacuum of 10 Pa and then flushed by air to a pressure of 60 Pa. After 120 s plasma treatment at 150 W, hydroxyl (–OH) groups were generated on the surface of the wings turning them hydrophilic. To avoid the wings floating on the surface of aqueous solutions and hindering subsequent experiments, both the dorsal and ventral sides of the wings were hydrophilized. To alter the main wing compound chitin to chitosan, a reduction process was carried out to remove inorganic salts. The wings were soaked in 1 M HCl for 2 h. Then, the wings were rinsed with double-distilled water to remove the consumed HCl. The wings were soaked in 2 M NaOH for 30 min. Then, the NaOH solution was heated to 80 °C (*M. menelaus*, 6 h; *P. u. telegonus* and *O. c. lydius*, 12 h). Finally, the wings were rinsed with double-distilled water twice to remove NaOH traces [46].

#### 2.3.2. Cell Culture

HepG2 cells (Drum Tower Hospital, Nanjing, China) were cultured in high-glucose Dulbecco’s modified Eagle’s medium (DMEM) supplemented with 10% fetal bovine serum with 2.5 mM l-glutamine, 0.5 mM sodium pyruvate, and 1200 mg/L sodium bicarbonate, 15 mM *N*-2-hydroxyethylpiperazine-*N*-2-ethanesulfonic acid, and 0.4 mg/mL G418 antibiotic (Invitrogen, Nanjing, China; Biotech Development Co., Ltd., Nanjing, China). The wings (substrates) were sterilized in 75% ethanol and 1 h ultraviolet irradiation. They were then placed into six-well culture plates. Sterilized metal rings were used to fix the substrate in the well to focus the cultured areas. The cells were seeded at a density of 1 × 10^5^ cells/mL and maintained at 37 °C in 5% CO_2_. The substrates were observed, and the culture medium was changed every two days until the cells were ready for imaging and other designed assessments.

#### 2.3.3. Methylthiazolyldiphenyl-Tetrazolium Bromide Viability Assay

The cells were cultured on the substrates for 48 h for acute toxicity, and for 2 h to 21 days for chronic toxicity. Cell viability was measured by MMT assay (Sigma, St. Louis, MO, USA). The tetrazolium ring is cleaved by mitochondrial dehydrogenase enzymes to form a purple precipitate. MTT (0.5 mg/mL) was added to wells in high-glucose DMEM without phenol red. After 1 h incubation, the purple precipitate was dissolved in a 1:1 solution of isopropanol and dimethyl sulfoxide (DMSO). The absorbance of the solution was recorded at 570 nm (SpectraMax spectrophotometer, Molecular Devices, Sunnyvale, CA, USA).

#### 2.3.4. Fluorescent Viability Staining 

To further assess their viability, the cells were incubated with 50 mM calcein acetoxymethyl ester (Calcein-AM, Molecular Probes, Eugene, OR, USA) for 20 min at 37 ◦C and stained cells were then visualized under a fluorescence microscope (OLYMPUS IX71, Tokyo, Japan) at 20× magnification. Digital images were acquired with a charge-coupled device (CCD) camera (Axiocam b/w, Zeiss). 

#### 2.3.5. Scanning Electron Microscopy

Cell cultures were fixed for 24 h at 20°C with 3% glutaraldehyde, then dehydrated using a graded series of ethanol of concentrations (25, 50, 70, 80, 90, 95, 100%), further dried using CO_2_ critical point dryer (HCP-2, Hitachi Ltd.), and sputter-coated with gold prior to scanning electron microscopy (SEM) imaging. The wings were also characterized using field emission scanning electron microscopy (FE-SEM) and EDS to confirm the element composition of the butterfly wings both before and after the acid/base treatment.

#### 2.3.6. Assessment of Hepatic Function

Albumin and urea secretion were determined by measuring the concentration of albumin and urea in the media. Albumin secretion was measured by ELISA using a rat albumin ELISA quantitation kit (Nanjing Jiancheng Bioengineering Institute), after 1, 2, 7, 15, and 21 days of culture. Urea synthesis was measured with commercially available kits (Nanjing Jiancheng Bioengineering Institute) using diacetyl monoxime. The absorbance was measured with a Quant microplate spectrophotometer (Elx-800, BioTek Instrument Inc.,) using purified rat albumin or urea dissolved in culture media (Nanjing Jiancheng Bioengineering Institute). Standard curves were generated.

#### 2.3.7. Statistical Analysis

All experiments were carried out for at least 2–3 times in duplicate or triplicate for each assessment. Data are expressed as the mean ± standard deviation. The statistical analyses used the Student’s *t*-test. We considered the value of *p* < 0.05 was statistically significant. Data were analyzed using SPSS version 11.0 (SPSS, Inc., Chicago, IL, USA).

## 3. Results and Discussion

**Figure 1:**
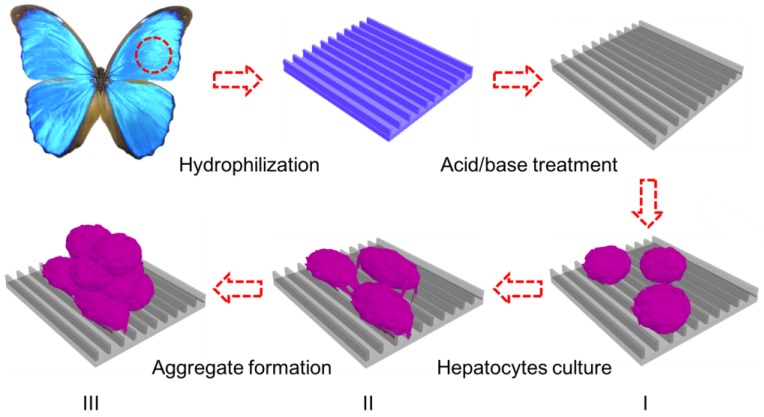
Schematic illustration of the in vitro three-dimensional (3D) model for hepatic culture and hepatocyte aggregate formation. Butterfly wings are chemically treated to turn them hydrophilic prior to HepG2 cell culture. (I) Fresh rat hepatocytes are dispersed onto treated butterfly wings; (II) Single hepatocytes begin migration and adhere together to form clusters; (III) Aggregate formation with extensive cell–cell communication and tight junctions.

Chitosan is used extensively in the clinic due to its biocompatibility and biodegradability, as well as its lack of immunoreactivity against cellular degradation products in vivo as compared to synthetic polymers [47]. It has been reported that cell–substrate interactions affect cell aggregate formation in culture, where the strength of cell–substrate adhesion directly affects cell morphology and direction of growth [48]. In our earlier studies, we developed a simple and green method utilizing substrates derived from butterfly wings with natural anisotropic microstructures. NIH-3T3 fibroblast cultured on these substrates showed a high degree of alignment along the direction of the grooves/ridges [45]. Here, we suggest that the chitin-based scaffolds naturally derived from butterfly wings may also have an impact on cell culture of the hepatoma HepG2 cells. We, thus, have established cell cultures on the chitin-based scaffolds naturally derived from butterfly wings and studied its impact on the growth of the hepatoma HepG2 cell line and formation of hepatocyte aggregates (Figure 1). Furthermore, we checked the liver-specific functions presented by testing the levels of albumin and urea secretion over 21 days.

**Figure 2:**
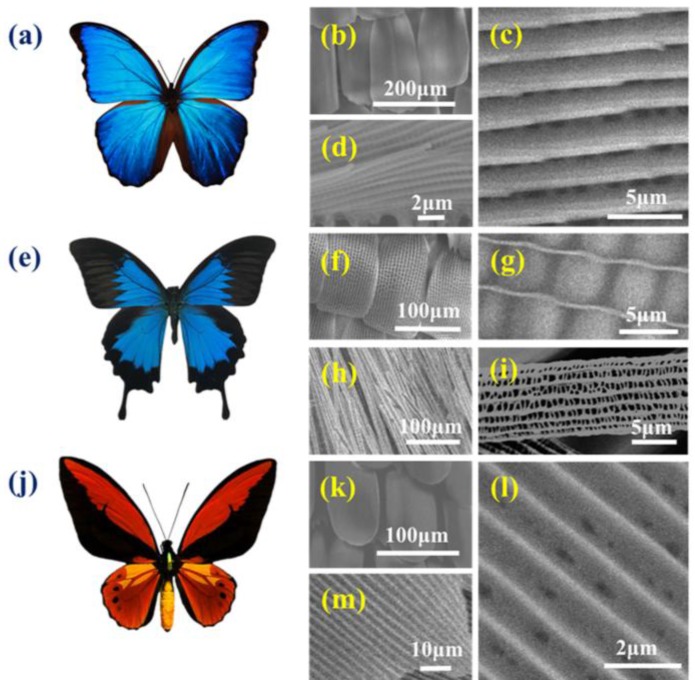
Topographical characterization of the butterfly wings. (**a**) Image of the *Morpho menelaus* butterfly; (**b**–**d**) scanning electron microscopy (SEM) images of the microstructure of the *M. menelaus* wing at different magnifications; (**e**) image of the *Papilio ulysses telegonus* butterfly; (**f**) SEM image of the microstructure of the blue area of the *P. u. telegonus* wing and (**g**) higher magnification SEM image; (**h**) SEM image of the microstructure of the fibrous area and (**i**) higher magnification SEM image; (**j**) image of *Ornithoptera croesus lydius* butterfly; (**k**–**m**) SEM images of the microstructure of the *O. c. lydius* wing at different magnifications.

Butterfly wings from the three species that were used in this study are characterized by distinct and different microstructures. Briefly, the *Morpho* wing structure is made up of multilayer ridges, whereas that of *O. c. lydius* is made up of vertical ridges, while *P. u. telegonus* wings are composed of two distinct microstructures. These distinct microstructures allow the transportation of light that moves through the photonic microstructure units composing the structural colors on the surface of the wings, beside the absorption of light by chemical chromophores that compose the coloring pigments [49]. Both structural colors and pigments provide the bright colors of these butterflies. The broad, flat scales that composed the *M. menelaus* wings (Figure 2a) has an anisotropic structure because of its microstructure on the surface made up of periodically ordered ribs and ridges, as shown in Figure 2b–d. The wings of the *P. u. telegonus* butterfly, which belongs to the *Papilio* genus, has two significantly distinct area: the blue area are groove/ridge structures that are similar to the anisotropic structures of the *M. menelaus* wings, while the black areas on the wings are composed of fibers (Figure 2e). The blue area composed of concaves and present ribbed ridges that appear to be smaller than those of *M. menelaus* wings. High magnification SEM images shows the details of the concaves in between the ridges (Figure 2f,g). The fibrous area is constructed of another photonic microstructure in the form of a nanofiber structure composed of rods formed by parallel fibers that consist of panes and ridges (Figure 2h,i). The wings of *O. c. lydius*, which belongs to the *Ornithoptera* genus, consist of yellow and black colors (Figure 2j). The yellow area’ microstructure also consists of parallel ridges, while the spaces between the ridges appear more “filled in” compared to *M. menelaus* wings (Figure 2k–m). These four anisotropic microstructures with their grooves/ridges and fibrous structure have been used in this study as a substrate for the in vitro HepG2 cell culture.

**Figure 3:**
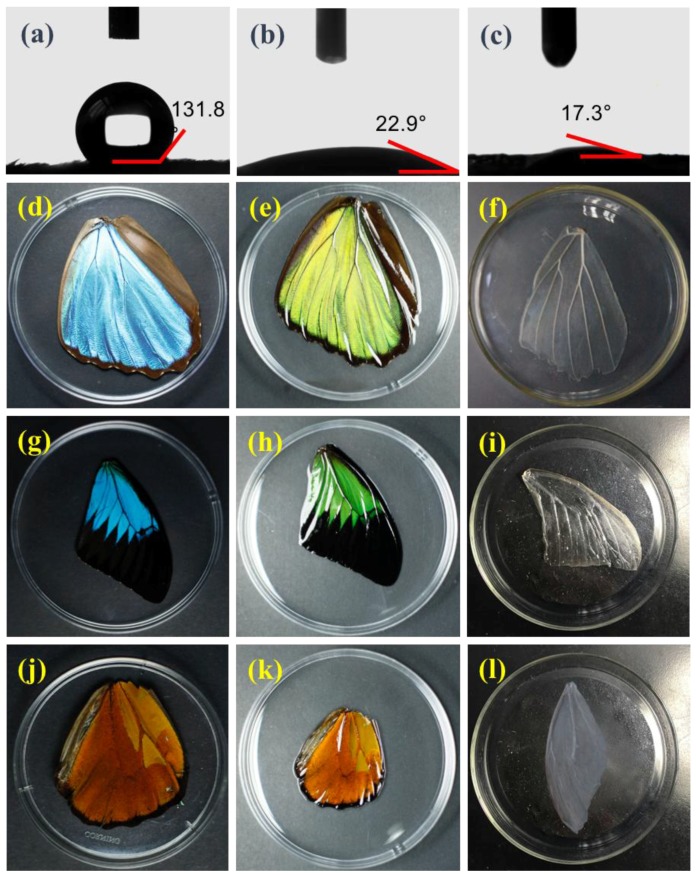
Treatment of butterfly wings. (**a**–**c**) Water contact angle on the wing surface of the *M. menelaus* butterfly wing (**a**) before and (**b**) after plasma treatment, and (**c**) after HCl/NaOH treatment. (**d**–**f**) Images of a *M. menelaus* wing (**d**) before and (**e**) after hydrophilic treatment, and (**f**) after HCl/NaOH treatment. (**g**–**i**) Images of a *P. u. telegonus* wing (**g**) before and (**h**) after hydrophilic treatment, and (**i**) after HCl/NaOH treatment. (**j**–**l**) Images of *O. c. lydius* wings (**j**) before and (**k**) after hydrophilic treatment, and (**l**) after HCl/NaOH treatment.

Following our standard protocol, two steps were applied to modify the wings prior to cell culture. First, the hydrophobicity of the butterfly wings was changed, and second, a chemical modification of the wings was performed to obtain more biocompatible substrates. To change the superhydrophobicity of the butterfly wings on both the dorsal and ventral sides of the wing surface, plasma treatment was applied to render hydrophilicity by generation of hydroxyl (–OH) groups on the wing surface, as shown in the water contact angle before and after treatment (Figure 3a,b). Butterfly wings were next treated with HCl/NaOH to remove pigments and other proteins that may affect cell viability and to get a more biocompatible compound for cell culture increasing further its wettability (Supplementary Figure S1 and Figure 3c). After treatment, *M. menelaus* wings kept their shiny blue color, although they turned transparent after HCl/NaOH treatment, indicating that the pigments were removed and the structural colors caused by the wing microstructure remained (Figure 3d–f). Wings from *P. u. telegonus* and *O. c. lydius* did not show any colors after the treatment, in support of the presence of pigment-based colors on the wing surface of these two-butterfly species (Figure 3g–l). Wings were then sterilized prior to cell culture, and hepatoma HepG2 cells were seeded on *M. menelaus*, *P. u. telegonus* (blue area), *P. u. telegonus* (fibrous area), *O. c. lydius* wings, and on a cell culture dish at a density of 1 × 10^5^ cells/mL. The experiments were carried out over three weeks. 

**Figure 4:**
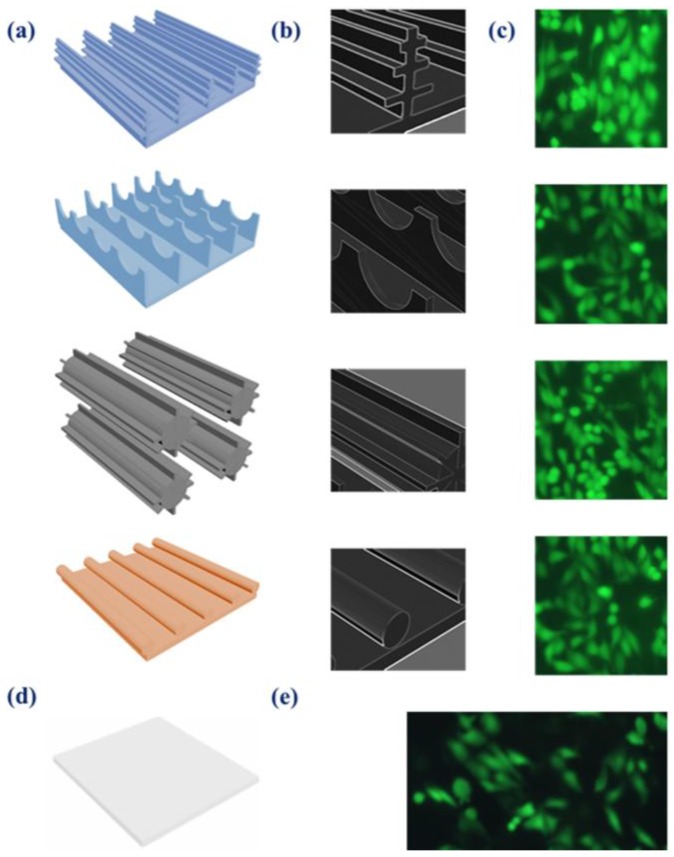
Aggregate formation on the selected natural anisotropic substrates. (**a**) Schematic of the anisotropic structures of the four substrates: *M. menelaus*, *P. u. telegonus* (blue area), *P. u. telegonus* (fibrous area), and *O. c. lydius* wings, from top to bottom, respectively. (**b**) Schematic focusing on the characteristic of the anisotropic structures of the four substrates: *M. menelaus*, *P. u. telegonus* (blue area), *P. u. telegonus* (f fibrous area), and *O. c. lydius* wings, from top to bottom, respectively. (**c**) Fluorescence microscopy images of HepG2 cells cultured on different substrates after 48 h: *M. menelaus*, *P. u. telegonus* (blue area), *P. u. telegonus* (fibrous area), and *O. c. lydius* wings, from top to bottom, respectively. (**d**) Schematic of the culture dish surface. (**e**) Fluorescence microscopy image of HepG2 cells cultured on a culture dish.

After 48 h of culture, Calcein-AM was used to stain the cells for fluorescence for a live cell viability imaging test. Cells growing on those butterfly wings showed a similar cell number compared to cells grown on the culture dish. This demonstrates that these chitin-based substrates derived from butterfly wings inherit its biocompatibility for hepatocyte culture (Figure 4).

**Figure 5:**
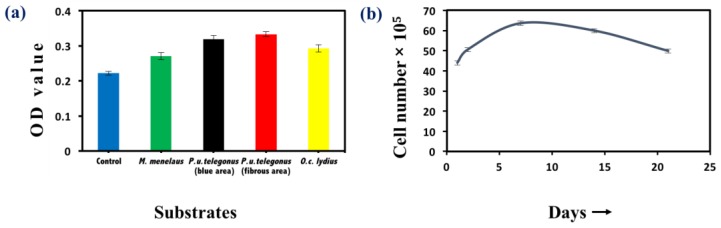
Cell viability assay. (**a**) Methylthiazolyldiphenyl-tetrazolium bromide (MTT) assay of HepG2 cells cultured on different substrates: *M. menelaus*, *P. u. telegonus* (blue area), *P. u. telegonus* (fibrous area), *O. c. lydius* wings, and cell culture dish (control) after 48 h. (**b**) Long period culture of HepG2 cells on a *P. u. telegonus* wing (fibrous area). Data are shown as mean ± standard deviation.

Cell viability was examined by MTT assay to examine cellular survival and measure the number of viable cells after 48 h and throughout the time course of these experiments. Cells seeded on *P. u. telegonus* wings (fibrous area) showed the highest viability, while cells cultured on wings from the other two species still showed high viability compared to cells seeded on a culture dish (Figure 5a). These results show that these naturally derived substrates are biocompatible for HepG2 growth. Through the 21 days of examination, growth of HepG2 cells on *P. u. telegonus* wings (fibrous area) reached the highest level at one week. Thereafter, cell viability declined over the course of the three-week period, but cells were still viable at 21 days (Figure 5b). Other substrates have also shown similar results (Supplementary Figure S2).

**Figure 6:**
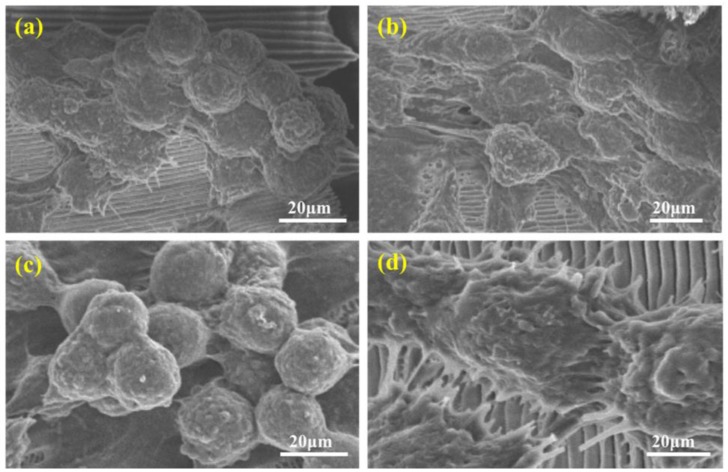
Scanning electron microscopy images of HepG2 cell aggregates after one week of culture on (**a**) *M. menelaus*, (**b**) *P. u. telegonus* (blue area), (**c**) *P. u. telegonus* (fibrous area), and (**d**) *O. c. lydius* wings.

Hepatocyte morphology and activity were examined for cells grown on each substrate. We chose day 7 as cells showed high viability by MTT assay to examine hepatocyte aggregate formation and function. Under the scanning electron microscope, HepG2 cells grown on *P. u. telegonus* wings (fibrous area) showed little cell–substrate contact area but formed spherical aggregates with extensive cell–cell contacts and tight junctions, and directional migration along the direction of aligned microfibers (Figure 6c). Similar results were shown for cells cultured on *P. u. telegonus* (blue area) and *M. menelaus* wings (Figure 6a,b). On *O. c. lydius* wings, the directional migration of hepatocytes was perpendicular to the direction of the ridges (Figure 6d). This may be because of the closer distance between the ridges and the reduced number of channels, and the network of pores compared to the multilayer and rich network of pores that compose the microstructures of the *P. u. telegonus* wing surface in both the blue and fibrous area and the wing of the *M. menelaus* butterfly.

**Figure 7:**
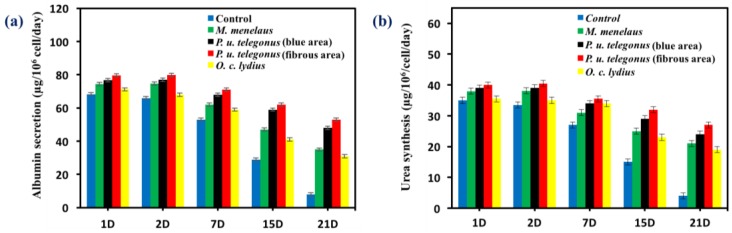
Liver-specific functions of hepatocytes cultured on different substrates during 21 days (D) of cell culture, including (**a**) albumin secretion and (**b**) urea synthesis. Data are shown as mean ± standard deviation.

Hepatocyte aggregates that formed on *P. u. telegonus* wings (fibrous area) showed excellent cellular activity, cell retention, and stable functional expression in terms of albumin secretion and urea synthesis activity compared to the monolayer culture of hepatocytes on the culture dish surface. Due to the formation of aggregates, hepatocytes cultured on *P. u. telegonus* wings (fibrous area) showed higher hepatic function over a 21-day culture period. Although levels of albumin secretion were maintained within the first two days, albumin secretion increased until day 7 and started to decline during the three weeks of the experiment time course. Similar results were observed for hepatocytes cultured on *P. u. telegonus* (blue area), *M. Menelaus*, and *O. c. lydius* wings, but in lower level compared to cells grown on the fibrous area of *P. u. telegonus* wings. Moreover, hepatocyte albumin secretion in the culture dish control experiment declined dramatically along the period of the experiment and on day 21, hepatocytes also lost their functions (Figure 7a). Similar results were observed for urea synthesis when hepatocytes were seeded on *P. u. telegonus* (fibrous area), *P. u. telegonus* (blue area), *M. menelaus*, *O. c. lydius* wings, and culture dish (Figure 7b). These results show that the anisotropic microstructure of our naturally derived substrates is biocompatible and have also a positive impact on hepatocyte growth, formation of aggregates, and function in vitro.

## 4. Conclusions

Here, we report a suitable in vitro 3D model for hepatocyte growth and aggregate formation maintaining the function of these cells taking advantage of the natural anisotropic microstructures derived from butterfly wings. These substrates, which we previously introduced as a simple, inexpensive, and green method, have been shown to be a suitable substrate for NIH-3T3 fibroblast cultured in vitro, promoting cell growth and a high degree of alignment along the direction of the ridges on the butterfly wing surface [45]. In this study, the microstructure of these natural substrates have been proved to play an important role in the final 3D organotypic model and hepatocyte aggregation. We have also shown that these natural, biodegradable, and biocompatible substrates promote the maintenance of a functional hepatic model with potential to be exploited for therapeutic application and regenerative tissue applications.

## Supplementary Materials

The following are available online at http://www.mdpi.com/2313-7673/3/1/2/s1, Figure S1: Energy-dispersive X-ray spectroscopy analysis of butterfly wings, Figure S2: Methylthiazolyldiphenyl-tetrazolium bromide assay on HepG2 cells cultured on butterfly wings.
