# Supplementary material for: Hepatocyte Aggregate Formation on Chitin-Based Anisotropic Microstructures of Butterfly Wings
Source: Biomimetics (Basel). 2018 Jan 18;3(1):2. doi: 10.3390/biomimetics3010002 (PMC6352657; doi:10.3390/biomimetics3010002)
Supplement: Supplementary file 1 [file biomimetics-03-00002-s001.pdf]

Biomimetics **2018**, *3*, 2

Supplementary Materials: Hepatocyte Aggregate Formation on Chitin-Based Anisotropic Microstructures of Butterfly Wings

Abdelrahman Elbaz [1,2], Bingbing Gao [1,2], Zhenzhu He [1,2], and Zhongze Gu [1,2,3,*]

1. State Key Laboratory of Bioelectronics, School of Biological Science and Medical Engineering, Southeast University, Nanjing 210096, China; chem.egy@gmail.com (A.E.); 230139435@seu.edu.cn (B.G.); 230169448@seu.edu.cn (Z.H.)
2. National Demonstration Center for Experimental Biomedical Engineering Education, Southeast University, Nanjing 210096, China
3. Laboratory of Environment and Biosafety Research Institute of Southeast University in Suzhou, Suzhou 215123, China

* Correspondence: gu@seu.edu.cn

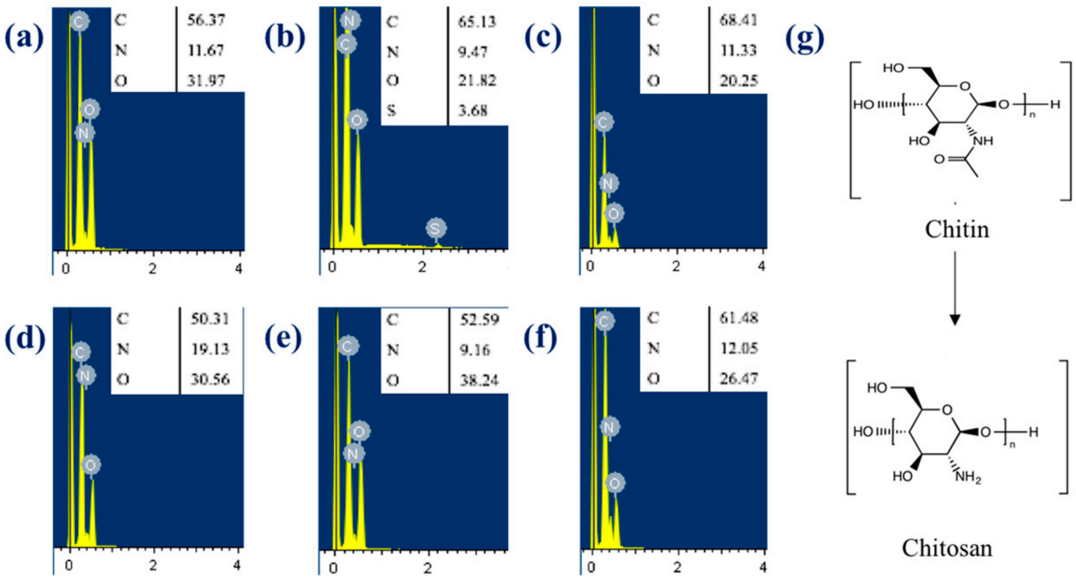

Figure S1. Energy-dispersive X-ray spectroscopy analysis of butterfly wings. *M. menelaus*, *P. u. telegonus* and *O. c. lydius* wings (**a**–**c**) after hydrophilic treatment and (**d**–**f**) after acid/base treatment, from left to right, respectively. (**g**) Schematic depicting the chemical composition changes on the butterfly wing surface throughout the treatment process.

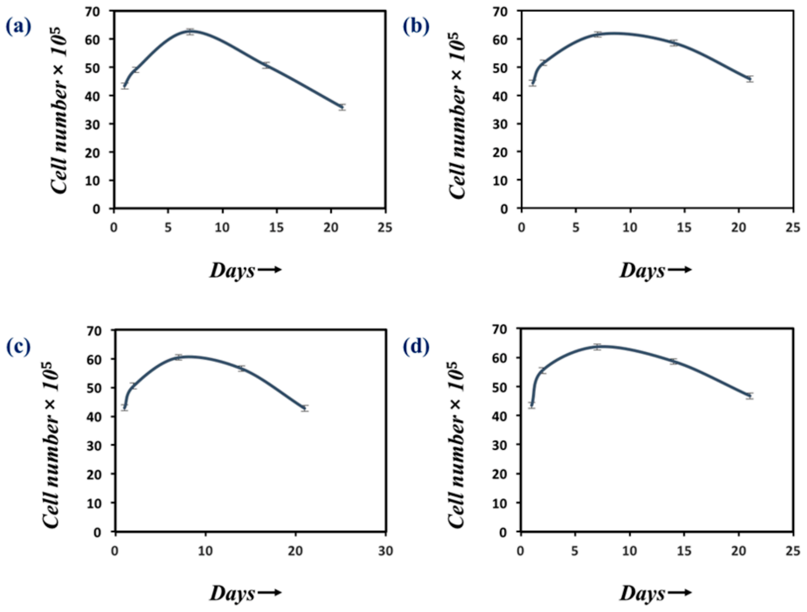

Figure S2. Methylthiazolyldiphenyl-tetrazolium bromide assay on HepG2 cells cultured on butterfly wings. (**a**–**d**) Number of cells cultured on different substrates: (**a**) *M. menelaus*, (**b**) *P. u. telegonus* (fibrous region), (**c**) *P. u. telegonu*s (blue region), and (**d**) *O. c. lydius* wings.
